# Updating Code of Ethics of the Psychiatric Association of Turkey: process and content

**DOI:** 10.1192/j.eurpsy.2024.85

**Published:** 2024-08-27

**Authors:** S. Vahip

**Affiliations:** ^1^Psychiatry, Ege University, Izmir, Türkiye

## Abstract

**Abstract:**

*Science, Ethics, Solidarity*… These three words are mottos of Psychiatric Association of Türkiye (PAT), since its foundation in 1995. In accordance, PAT has Code of Ethics for more than 20 years. There are many developments and changes both in practicing psychiatry and in the community in the last couple of decades. As a result, many new ethical questions, dilemmas and approaches arise for psychiatry. PAT had decided to update the Code of Ethics about two years ago and the updating process is almost on the edge of finalization.

In this presentation, main points of the updated and newly written principles will be summarized with special references to recent developments in the world and updated or newly written international Code of Ethics such as, EPA, WPA, and several national associations’ documents.

One of the most important outcome and benefit of such an updating process is modelling the project on a participatory base, having wide feedback and inputs from experts (as many as possible, enriched by diverse interests of related disciplines) and reaching as many colleagues as possible from different working conditions. A six step project with this perspective was prepared and implemented, and will be summarized in the presentation for international exchange of experiences.

**Disclosure of Interest:**

None Declared

